# Efficacy and Predictors for Biofeedback Therapeutic Outcome in Patients with Dyssynergic Defecation

**DOI:** 10.1155/2017/1019652

**Published:** 2017-08-29

**Authors:** Ting Yu, Xiaoxue Shen, Miaomiao Li, Meifeng Wang, Lin Lin

**Affiliations:** ^1^Department of Gastroenterology, The First Affiliated Hospital of Nanjing Medical University, No. 300 of Guangzhou Road, Nanjing, China; ^2^Department of Gastroenterology, Huaian First Hospital Affiliated to Nanjing Medical University, No. 6 of West Beijing Road, Huaian, China

## Abstract

**Aim:**

To evaluate the short-term efficacy of biofeedback therapy (BFT) for dyssynergic defecation (DD) and to explore the predictors of the efficacy of BFT.

**Methods:**

Clinical symptoms, psychological state, and quality of life of patients before and after BFT were investigated. All patients underwent lifestyle survey and anorectal physiology tests before BFT. Improvement in symptom scores was considered proof of clinical efficacy of BFT. Thirty-eight factors that could influence the efficacy of BFT were studied. Univariate and multivariate analysis was conducted to identify the independent predictors.

**Results:**

Clinical symptoms, psychological state, and quality of life of DD patients improved significantly after BFT. Univariate analysis showed that efficacy of BFT was positively correlated to one of the 36-item Short-Form Health Survey terms, the physical role function (*r* = 0.289; *P* = 0.025), and negatively correlated to the stool consistency (*r* = −0.220; *P* = 0.032), the depression scores (*r* = −0.333; *P* = 0.010), and the first rectal sensory threshold volume (*r* = −0.297; *P* = 0.022). Multivariate analysis showed depression score (*β* = −0.271; *P* = 0.032) and first rectal sensory threshold volume (*β* = −0.325; *P* = 0.013) to be independent predictors of BFT efficacy.

**Conclusion:**

BFT improves the clinical symptoms of DD patients. Depression state and elevated first rectal sensory threshold volume were independent predictors of poor outcome with BFT.

## 1. Introduction

Chronic constipation (CC) is diagnosed when there is at least a 6-month history of symptoms such as infrequent bowel movement, reduced stool volume, hard stools, and excessive straining at defecation [1]. Treatment can be very difficult. The median prevalence is 16% in the US and is as high as 33.5% in adults aged 60–101 years [2]. The overall prevalence in Chinese adults is 16%–20% [3].

Primary constipation consists of several overlapping subtypes, among which dyssynergic defecation (DD) is relatively common [4, 5]. Patients with DD have symptoms of obstructive defecation, such as severe straining during defecation and a sensation of a “blockage” and of incomplete evacuation. The physiological mechanisms of DD include inability to coordinate abdominal, rectoanal, and pelvic floor muscles during defecation because of causes such as inadequate rectal and/or abdominal propulsive force, impaired anal relaxation (i.e., <20% relaxation of basal resting pressure), or increased anal outlet resistance as a result of paradoxical external anal sphincter or puborectalis contraction [6, 7]. Pharmacological therapies that are usually effective in CC, such as bulking agents, osmotic laxatives, stimulant laxatives, and stool softeners [8], are often ineffective in DD patients [9].

Biofeedback therapy (BFT), which is based on behavior modification [10], can be used to train DD patients to defecate effectively. Patients are taught to brace the abdominal wall muscles and relax the pelvic floor muscles during defecation, and efforts are also made to modify sensory perception in the rectum [11]. The first application of BFT for treatment of CC due to DD was in 1987 [12]. Since then, a number of controlled studies have shown that BFT can be more effective than laxatives, muscle relaxants, and placebo, with benefits lasting for at least 12 months [13–15]. Based on these findings, BFT has been recognized as the most effective treatment for DD for several years [16, 17]. However, symptomatic improvement after BFT has varied widely between studies, ranging from 44% to 100% [18]. Few data are available regarding the factors predictive of success of BFT [19]. In our experience, we have seen that anorectal physiology, psychological state, quality of life, and lifestyle factors can all influence the efficacy of BFT.

The aim of this study was to investigate the short-term efficacy of BFT and to identify the clinical and physiological factors that predict success or failure following BFT in Chinese patients.

## 2. Material and Methods

### 2.1. Patients

In this retrospective study, all adult patients diagnosed with CC due to DD at the Department of Gastroenterology of the First Affiliated Hospital of Nanjing Medical University, between January 1, 2012, and October 30, 2015, were eligible for inclusion. CC was diagnosed if the patient had at least two of the following constipation symptoms for >6 months: (1) infrequent stools (<3 bowel movements/week); (2) hard or lumpy stools (Bristol stool form scale score of 1-2) [20]; (3) straining at stool; (4) sensation of incomplete evacuation after bowel movement; or (5) sensation of anorectal blockage [21]. The presence of DD was determined using high-resolution anorectal manometry (HR-ARM) and rectal balloon expulsion test. Patients presented with inappropriate contraction or inadequate propulsive forces in HR-ARM and prolonged balloon expulsion time were considered to have DD. None of the patients had responded to standard management of constipation (e.g., increased dietary fiber and fluid intake or laxatives). Patients were excluded from the study if they (1) were <18 years in age, (2) had structural bowel disease or history of abdominal surgery, (3) had mental illness, (4) had recently received psychotropic drugs [22], (5) were pregnant, or (6) had not completed a full course of BFT (4 sessions).

This study was approved by the Ethics Committee of the First Affiliated Hospital of Nanjing Medical University (2016-SRFA-064).

### 2.2. Constipation Severity

A questionnaire (Table 1) adapted from the one developed by the Cleveland Clinic was used to assess defecatory symptoms [23] such as frequency of spontaneous bowel movements, stool consistency, straining during defecation, sensation of incomplete evacuation, sensation of blockage, and painful defecation. The latter four are deemed to be relatively specific for DD and were scored on a scale of 0 to 3, where 0 = never occurred, 1 = occurred occasionally, 2 = occurred during 25% of defecations, and 3 = occurred during 50% of defecations. The frequency of spontaneous bowel movements was scored as 0 = defecation interval 1-2 days, 1 = defecation interval 3 days, 2 = defecation interval 4-5 days, and 3 = defecation interval > 5 days. Stool consistency was evaluated according to the Bristol stool scale (a 7-point scale, ranging from 1 = separate hard lumps like nuts to 7 = watery) [20]; in this study, the scores were allotted as follows: Bristol type 4–7 = score 0, Bristol type 3 = score 1, Bristol type 2 = score 2, and Bristol type 1 = score 3.

### 2.3. Assessment of Psychological State and Quality of Life

Zung's Self-Rating Anxiety Scale (SAS) [24] and Self-Rating Depression Scale (SDS) [25] were used to evaluate the levels of anxiety and depression. In Chinese populations, SAS ≥ 50 and SDS ≥ 53 represent diagnosable anxiety and depression [26]. The 36-item Short-Form Health Survey (SF-36) was used to evaluate quality of life [27]. The SF-36 consists of eight sections: vitality, physical functioning, bodily pain, general health perceptions, physical role functioning, emotional role functioning, social role functioning, and mental health. The scores in each section are the weighted sums of the scores for each question in that section. The scale is directly transformed into a 0–100 scale on the assumption that each question carries equal weight. The higher the score, the better the patient's quality of life.

### 2.4. Lifestyle Survey

Information on physical activity, work pressure, and sleep quality were obtained from questionnaires filled in at first contact with the patient. Physical activity was assessed by one question on the frequency of exercise of at least 30 minutes per session during the past week; the possible responses were “often,” “sometimes,” “seldom,” and “never.” Work pressure was graded as “low,” “normal,” high,” and “very high.” Sleep quality was assessed by the Pittsburgh Sleep Quality Index (PSQI) questionnaire [28]. The PSQI assesses seven components of sleep: the quality, latency, duration, and efficiency of sleep, sleep disturbances, use of sleeping medication, and daytime dysfunction. Each component is scored from 0 to 3, and the seven component scores are summed to gain a global score. In Chinese populations, a PSQI global score > 7 indicates poor sleep quality [29].

There were six questions on the frequency and/or volume of consumption of certain food items. Volume of intake was graded as “low,” “normal,” “high,” or “very high,” and frequency of consumption as “often,” “sometimes,” “seldom,” or “never.” Thus, we recorded the frequency and volume of consumption of vegetables (seldom, 250–<500 g/d, 500–1000 g/d, and >1000 g/d); fruits (seldom, 100–200 g/d, 200–500 g/d, and >500 g/d); and water (<500 mL/d, 500–1000 mL/d, >1000 mL/d). Predilection for a high-fat diet was also recorded (yes/no).

### 2.5. Rectal Balloon Expulsion Test

The time required for subjects to expel a rectal balloon filled with 50 mL of warm water while seated in privacy on a commode was measured. The balloon was removed if the subject was not able to expel the balloon within 1 minute [30, 31].

### 2.6. Colonic Transit Study

Colonic transit was assessed using radiopaque marker techniques. In brief, the patient ingested a single capsule containing 24 cylindrical radiopaque markers of 2 mm diameter and 6 mm length on day 1. A supine radiograph of the abdomen was obtained on day 3 (i.e., 72 hours later) to assess the number and distribution of the markers in the colon; patients were deemed positive for delayed colonic transit if there were >4 markers distributed throughout the colon [32, 33].

### 2.7. High-Resolution Anorectal Manometry

A novel solid-state HR-ARM device (Manoscan AR 360; Given Imaging, Yokneam, Israel) with 12 sensors was used for anorectal manometry. The procedure was performed after defecation. The patient was placed in the left lateral decubitus position, with the hips flexed to 90°. The rectal balloon, with the attached catheter, was placed 3 cm proximal to the upper part of the anal sphincter. Measurements were made in the following order: resting anal and rectal pressure (20–30 seconds), pressure during squeeze (best of three attempts, with a maximum duration of 20–30 seconds per attempt), and pressure during bearing down as in defecation (best of three attempts, with 20–30 seconds per attempt) [34]. Rectal sensation was simultaneously evaluated; for this, the rectal balloon was progressively distended in 10 mL increments from 0 mL to 50 mL, and threshold volumes for first sensation, urgency, and maximum discomfort were recorded.

Four phenotypes of DD have been recognized based on HR-ARM: type I dyssynergia, in which there is an adequate increase (≥40 mmHg) in rectal pressure, accompanied by a paradoxical simultaneous increase in anal pressure; type II dyssynergia, in which there is an inadequate increase (<40 mmHg) in rectal pressure (poor propulsive force), accompanied by a paradoxical increase in anal pressure; type III dyssynergia, in which there is an adequate increase (≥40 mmHg) in rectal pressure, accompanied by failure of reduction in anal pressure (to ≤20% of baseline pressure); and type IV dyssynergia, in which there is an inadequate increase (<40 mmHg) in rectal pressure (poor propulsive force), accompanied by failure of reduction in anal pressure (to ≤20% of baseline pressure) [1].

### 2.8. Biofeedback Training

The Polygraf ID 8 (Medtronic Ltd, Denmark) was used for biofeedback training. Patients received a 1-hour biofeedback training once every other day for the first 2 weeks, and 2-3 times per week thereafter. For the training session, the patient was asked to lie on the right side, and a single manometry catheter and anal electrode were inserted into the patient's anorectal canal at the sphincter. The catheter and the electrode were connected to the Polygraf ID, which displayed the data collected in the anorectal canal in a simple graphical format. The biofeedback application displayed a column, which the patient navigated using the pelvic floor muscles. By contracting and relaxing the pelvic floor muscles, the patient could move the signal level indicator up and down. The patient was instructed to try and keep the signal level within the limits of the column, while maintaining awareness of the changes in the pelvic floor muscle activity. They could thus learn to modulate the activity of the anorectal muscles [35]. During the training period, patients were required to practice at home, using the squeezing and relaxing maneuvers for 20 minutes at a time, 2-3 times/week. At the conclusion of biofeedback training, all patients were told that their pushing efforts had improved; this ensured that patients would be motivated to return for a follow-up and have positive expectations during the follow-up assessments.

### 2.9. Evaluation of Biofeedback Treatment Efficacy

Treatment efficacy was assessed at the completion of the BFT session. Treatment efficacy was expressed as a ratio, that is, the difference between the pretraining and posttraining constipation severity scores divided by the pretraining score, and graded as “very efficacious” (score > 0.05), “efficacious” (score 0.25–0.50), or “not efficacious” (score < 0.25).

### 2.10. Statistical Analysis

All data were analyzed using SPSS version 20.0 (IBM Corp., Armonk, NY, USA). Continuous variables were expressed as means ± standard deviation or medians (range), and categorical variables as relative frequencies. Student's *t*-test or the Mann–Whitney *U* test was used to compare continuous variables, and the chi-square test or Fisher's exact test for categorical variables. Univariate and multivariate analysis was used to identify the predictors of BFT efficacy. *P* < 0.05 was considered statistically significant.

## 3. Results

The data of 171 patients (69 men and 102 women; mean age, 54.0 ± 23.3 years) were analyzed.

### 3.1. Baseline Clinical Symptoms, Psychological State, and Quality of Life

The mean disease duration was 6.5 ± 2.5 years. In this study population, 74.9% (128/171) patients had not had spontaneous bowel movements over the past 2 years. In all, 93.0% (159/171) patients had history of long-term use of stimulant laxatives. The mean defecation interval was 1.95 ± 1.20 days, and the mean stool consistency score was 1.82 ± 1.20. Almost all patients had complaints of straining during bowel movement, sensation of incomplete defecation, sensation of blockage, or pain during defecation.  Table 2 shows the defecatory symptom scores.

The anxiety and depression scores were 40.0 ± 15.5 and 50.1 ± 13.5, respectively, which were significantly higher than the Chinese norms (33.80 ± 5.90 and 41.88 ± 10.57, resp.; Table 3) [26]; on the basis of these scores, 22.2% (38/171) and 33.9% (62/171) of the patients had anxiety and depression, respectively.

Table 4 shows the scores of the DD patients in the different sections of the SF-36. All scores were significantly lower than the Chinese norms [36].

### 3.2. Baseline Lifestyle Factors

Table 5 shows the scores for physical activity, work pressure, sleep quality, and diet habits of the DD patients before BFT.

### 3.3. Baseline Anorectal Physiology

In this study, 48.5% (83/171) patients presented with prolonged colonic transit time. The mean values for the manometric parameters were as follows: anal resting pressure 82.5 ± 16.0 mmHg, maximum squeeze pressure 208.3 ± 41.5 mmHg, rectal defecation pressure 38.9 ± 8.6 mmHg, intrarectal pressure 88.9 ± 15.3 mmHg, and rectoanal pressure differential −42.0 ± 8.5 mmHg, and threshold for the first sensation 60.0 mL (range, 20.0–220.0 mL), urgency 100.0 mL (range, 40.0–350.0 mL), and maximum discomfort 150.0 mL (range, 80.0–350.0 mL). According to the HR-ARM results, 82/171 (48.0%), 51/171 (29.8%), 30/171 (17.5%), and 8/171 (4.7%) patients were classified as type I, type II, type III, and type IV DD, respectively.

### 3.4. Biofeedback Treatment Efficacy

Patients in this study received 10.0 ± 3.5 sessions of BFT. Treatment was assessed as “very efficacious” in 72.5% (124/171) patients, as “efficacious” in 8.2% (14/171) patients, and as “not efficacious” in 19.3% (33/171) patients; thus, the total efficacy was 80.7% (Table 6).

There was a very significant decrease in the total and subscale scores of bowel symptoms (defecation interval, straining at defecation, sensation of incomplete evacuation/blockage, and stool consistency; Table 2).

Anxiety and depression was markedly improved, with significant decrease in the scores of SAS and SDS after the BFT (Table 3). In the SF-36, the scores for general health perception, physical functioning, and bodily pain were increased significantly, indicating improvement in quality of life (Table 4).

### 3.5. Predictors of Outcome of BFT

Tables 7 and 8 show the association between the efficacy of BFT and psychological state, quality of life, lifestyle factors, and anorectal physiology. Univariate analysis showed that BFT efficacy was positively correlated to the score for physical role function (*r* = 0.289; *P* = 0.025) and negatively correlated to the stool consistency score (*r* = −0.220; *P* = 0.032), the depression score (*r* = −0.333; *P* = 0.010), and the first sensory threshold volume (*r* = −0.297; *P* = 0.022; Table 7). Multivariate analysis showed that depression score (*β* = −0.271; *P* = 0.032) and the first sensory threshold volume (*β* = −0.325; *P* = 0.013) were independent predictors of BFT efficacy (Table 8).

## 4. Discussion

In this study, we evaluated the efficacy of BFT in DD and attempted to identify the factors that could predict the success of BFT. We found that BFT could improve the clinical symptoms of patients with DD. The psychological state and the rectal first sensory threshold pressure were independent predictors of BFT outcome.

The prevalence of anxiety and depression in DD patients was much higher than the rates in the general population. These findings are consistent with previous literature that has documented a positive association—though not a causal relationship—between certain psychological disorders and DD [37, 38]. We found that DD patients also have lower quality of life than the general population. This is not surprising, as the symptoms of constipation and psychological disorders can both disrupt daily living.

DD patients in our study frequently experienced excessive straining at defecation and a sensation of incomplete evacuation, with the average scores of >2 indicating that symptoms occurred during at least 25% of defecations. Prolonged colonic transit time was seen in 49.2% of DD patients. Significant overlap (10%–60%) between slow-transit constipation and DD as well as between slow-transit constipation and constipation-predominant irritable bowel syndrome has been described previously [39, 40], which suggests that a proportion of patients with constipation may have colonic motor and/or sensory dysfunction and coexisting anorectal sensorimotor dysfunction. In our study population, type I dyssynergia was seen in 48.6%, type II dyssynergia in 28.4%, type III dyssynergia in 20.8%, and type IV dyssynergia in 2.1% of the patients. These rates are consistent with previous studies [6, 41]. The pathogenic mechanisms are different for the different subtypes of DD, and the response to BFT may vary greatly between subtypes.

Recent controlled studies have shown that BFT is an effective treatment for pelvic floor dyssynergia [15, 42, 43]; BFT was found to be superior to laxatives, with improvement being maintained over a long-term follow-up. The superior efficacy of BFT was also demonstrated by Wang et al. [44] in their study of 50 CC patients. Seventy percent of their patients felt that BFT was helpful, and 62.5% were improved. Clinical manifestations such as straining, abdominal pain, and bloating were relieved, and the use of oral laxative decreased after BFT; frequency of spontaneous bowel movement and psychological state were also improved significantly after BFT. In our study, at the end of training, there was significant decrease in total and subscale scores of clinical symptoms, including frequency of spontaneous bowel movement, straining at defecation, sensation of incomplete evacuation, sensation of blockage, and stool consistency, suggesting that BFT was an effective behavioral treatment for DD. The emotional centers in the brain can affect motility and sensation in the gut, acting mainly via the hypothalamic-hypophyseal axis and brain-gut axis. Studies have shown that depression increases pelvic floor muscle tension and reduces rectal sensitivity [45, 46]. Mild depression can be relieved to some extent by psychological counseling and by explanation of the symptoms. Both of these approaches are components of BFT, and therefore, BFT can improve the symptoms of both constipation and depression and help improve the overall quality of life of DD patients.

In our study, a harder stool was predictive of a substantial improvement in defecation symptoms after BFT. This finding is not unexpected because hard stool is a common feature of DD [1] and because BFT is known to improve dyssynergia and allow more efficient stool evacuation. Shim et al. studied 102 patients with CC and reported similar findings [47].

The SDS score was another predictor of BFT efficacy. Many patients with chronic diseases have concurrent depression. Depression is associated with poor treatment compliance, and some researchers consider that this may be an important factor for the failure of BFT in some patients [48–50]. In addition, patients with depression have autonomic nervous dysfunction; low vagal tone can result in decreased gastrointestinal motility [51]. However, Ding et al. have demonstrated that BFT has no effect on autonomic nervous function [35].

In our study, the only physiological parameter predictive of substantial improvement in defecation after BFT was the rectal first sensory threshold volume, an elevated value being related to poorer outcome with BFT. There could be several mechanisms for this. Normal rectal sensory function is essential for normal defecation. Patients with rectal hyposensitivity have elevated sensory thresholds, with resulting rectal dysfunction. Fecal retention in the rectum resulting from decreased desire to defecate leads to absorption of moisture from the stool, making it dry and hard. In addition, Schouten et al. have shown that rectal hyposensitivity patients have lower rectal contractility in response to rectal dilatation than control patients [52]. Decreased colonic motility could be another reason. Some rectal hyposensitivity patients have a primary decrease in colonic motility. Chronic dilatation of the rectum in these patients can cause a secondary decrease in proximal colonic motility (the rectum-colon reflex) [53]. Although there are studies proving the efficacy of BFT in slow-transit constipation, the findings are still debated [11, 54]. Currently, there is no effective therapy available for rectal hyposensitivity; the options include sensory training, neural regulation, and surgery.

The results of this study may have been affected by some limitations. First, there is no uniform criterion for the curative effect of BFT on DD. We use the valid score that equals the decreasing index between pretraining and posttraining constipation severity scores divided by pretraining score to assess the efficacy. The constipation severity score used in our study was made up of the duplicate entries of the Cleveland Clinic Constipation Score and Rome III criteria. However, we have not test the reliability and validity of the questionnaire. This questionnaire may not reflect the constipation symptoms of the patient accurately. Second, BFT efficacy was assessed at the completion of the BFT session. We have not assessed the long-term outcome of BFT. Also, we do not know the predictors for long-term efficacy of BFT.

## 5. Conclusion

BFT improves the clinical symptoms of DD patients. High SDS score and elevated first rectal sensory threshold volume are independent predictors of poor outcome with BFT. Treatment for depression and rectal hyposensitivity could optimize the effects of BFT in DD patients.

## Figures and Tables

**Table 1:** Scoring system for symptoms of DD.

Grading/score	Defecation interval (days)	Straining	Sensation of incomplete evacuation	Sensation of blockage	Painful defecation	Stool consistency
0	1-2	None	None	None	None	BSS: 4–7
1	3	Occurs occasionally	Occurs occasionally	Occurs occasionally	Occurs occasionally	BSS: 3
2	4-5	Occurs during >25% of defecations	Occurs during >25% of defecations	Occurs during >25% of defecations	Occurs during >25% of defecations	BSS: 2
3	>5	Occurs during >50% of defecations	Occurs during >50% of defecations	Occurs during >50% of defecations	Occurs during >50% of defecations	BSS: 1

DD = dyssynergic defecation; BSS = Bristol stool scale.

**Table 2:** Symptom scores before and after BFT.

Clinical symptoms	Before BFT	After BFT	*P*
Defecation interval (days)	1.95 ± 1.20	1.20 ± 0.91	0.039
Straining	2.75 ± 1.63	1.60 ± 1.15	0.042
Sensation of incomplete evacuation	2.50 ± 1.35	1.62 ± 1.15	0.048
Sensation of blockage	1.82 ± 1.40	0.95 ± 1.07	0.021
Painful defecation	1.20 ± 0.90	1.17 ± 074	0.109
Stool consistency	1.82 ± 1.20	0.96 ± 1.13	0.034
Total	12.36 ± 6.00	7.61 ± 4.52	0.011

Data are expressed as mean ± standard deviation. BFT = biofeedback therapy.

**Table 3:** SAS and SDS scores before and after BFT.

	Before BFT	After BFT	*P*
SAS	40.0 ± 15.5	33.5 ± 10.9	0.004
SDS	50.1 ± 13.5	46.0 ± 13.5	0.023

Data are expressed as mean ± standard deviation. SAS = Zung's Self-Rating Anxiety Scale; SDS = Zung's Self-Rating Depression Scale; BFT = biofeedback therapy.

**Table 4:** Scores for different quality of life indicators before and after BFT.

Quality of life indicator	Before BFT	After BFT	*P*
General health perception	41.3 ± 19.0	63.4 ± 19.2	<0.001
Physical functioning	84.0 ± 42.8	88.5 ± 39.2	0.045
Physical role functioning	60.5 ± 34.9	72.6 ± 39.0	0.033
Emotional role functioning	63.8 ± 32.0	75.4 ± 37.3	0.038
Social role functioning	74.0 ± 37.7	80.1 ± 37.5	0.087
Bodily pain	75.0 ± 40.0	86.3 ± 36.9	0.029
Vitality	62.1 ± 30.5	70.8 ± 23.0	0.040
Mental health	63.2 ± 23.6	65.9 ± 21.0	0.049

Data are expressed as mean ± standard deviation. BFT = biofeedback therapy.

**Table 5:** Frequency table of lifestyle characteristics.

Characteristic	Frequency (*n*)	Characteristic	Frequency (*n*)
Physical activity		Fruit intake	
Often	41	Seldom	11
Sometimes	67	100–200 g/d	90
Seldom	57	200–500 g/d	60
Never	6	>500 g/d	10
Work pressure		Water intake	
Low	106	<500 mL/d	53
Normal	30	500–1000 mL/d	100
High	26	>1000 mL/d	18
Very high	9	High-fat diet predilection	
Poor sleep quality		Yes	57
No	118	No	114
Yes	53		
Vegetable intake			
Seldom	19		
250–<500 g/d	44		
500–1000 g/d	67		
>1000 g/d	41		

**Table 6:** Clinical efficacy of BFT.

Clinical efficacy	*n*	Proportion (%)	Grading of efficacy (%)
≥75%	73	42.7%	Very efficacious (72.5%)
50%–75%	51	29.8%	
25%–50%	14	8.2%	Efficacious (8.2%)
≤25%	33	19.3%	Not efficacious (19.3%)

BFT = biofeedback therapy.

**Table 7:** Univariate analysis of predictors of outcome of BFT.

Variables	*r*	*P*
General information		
Age	−0.095	0.440
Gender	−0.112	0.202
Constipation duration	0.115	0.197
Symptoms		
Defecation interval	−0.062	0.683
Straining	−0.121	0.149
Sensation of incomplete evacuation	−0.092	0.450
Sensation of blockage	−0.145	0.106
Painful defecation	−0.040	0.849
Stool consistency	−0.220	0.032
Psychological status		
SAS	−0.184	0.093
SDS	−0.333	0.010
Quality of life indicators		
General health perception	0.135	0.116
Physical functioning	0.112	0.202
Physical role function	0.289	0.025
Emotional role functioning	0.120	0.207
Social role functioning	0.153	0.104
Bodily pain	0.046	0.751
Vitality	0.196	0.084
Mental health	0.205	0.057
Lifestyle		
Physical activity	−0.079	0.666
Work pressure	−0.089	0.490
Poor sleep quality	−0.078	0.666
Vegetable intake	0.145	0.106
Fruit intake	−0.062	0.683
Water intake	−0.095	0.468
High-fat diet predilection	0.017	0.800
Anorectal physiology		
BET time	−0.188	0.091
CTT	−0.062	0.711
HR-ARM		
Anal resting pressure	0.066	0.705
Maximum squeeze pressure	−0.030	0.761
Rectal defecation pressure	0.082	0.650
Intrarectal pressure	0.044	0.795
Rectoanal pressure differential	0.197	0.090
First sensation volume	−0.297	0.022
Urgency volume	−0.178	0.091
Maximum discomfort volume	−0.074	0.700
DD subtype	−0.099	0.365

BFT = biofeedback therapy; SAS = Zung's Self-Rating Anxiety Scale; SDS = Zung's Self-Rating Depression Scale; BET = balloon expulsion test; CTT = colonic transit time.

**Table 8:** Multiple linear regression analysis of predictors of BFT outcome.

Variables	*β* coefficient	95% CI	*P*
Stool consistency	−0.110	−0.213 to −0.032	0.176
SDS	−0.271	−0.506 to −0.036	0.032
Physical role function	0.112	0.204 to 0.020	0.172
First sensation volume	−0.325	−0.534 to −0.012	0.013

BFT = biofeedback therapy; SDS = Zung's Self-Rating.

## Abbreviations

BFT: Biofeedback therapy
DD: Dyssynergic defecation
CC: Chronic constipation
HR-ARM: High-resolution anorectal manometry
SAS: Zung's Self-Rating Anxiety Scale
SDS: Zung's Self-Rating Depression Scale
SF-36: 36-item Short-Form Health Survey
PSQI: Pittsburgh Sleep Quality Index.
